# Twins from different fathers: A heteropaternal superfecundation case report in Colombia

**DOI:** 10.7705/biomedica.5100

**Published:** 2020-12-09

**Authors:** Fernanda Mogollón, Andrea Casas-Vargas, Fredy Rodríguez, William Usaquén

**Affiliations:** 1 Grupo de Genética de Poblaciones e Identificación, Instituto de Genética, Universidad Nacional de Colombia, Bogotá, D.C., Colombia Universidad Nacional de Colombia Grupo de Genética de Poblaciones e Identificación Instituto de Genética Universidad Nacional de Colombia BogotáD.C Colombia

**Keywords:** DNA fingerprinting, paternity, twins, dizygotic, fertilization, microsatellite repeats, dermatoglifia del ADN, paternidad, gemelos dicigóticos, fertilización, repeticiones de microsatélite

## Abstract

Heteropaternal superfecundation is an extremely rare phenomenon that occurs when a second ova released during the same menstrual cycle is additionally fertilized by the sperm cells of a different man in separate sexual intercourse.

In August, 2018, the *Grupo de Genética de Poblaciones e Identificación* at *Universidad Nacional de Colombia* received a request to establish the paternity of a pair of male twins with genetic markers. The following analyses were performed: amelogenin gene, autosomal short tandem repeat (STR), and Y-STR analyses by means of human identification commercial kits, paternity index, and the probability of paternity calculation and interpretation. A paternity index of 2.5134E+7 and a probability of paternity of 99.9999% for twin 2 were obtained while 14 out of 17 Y-chromosome markers and 14 out of 21 autosomal short tandem repeats were excluded for twin 1. The results indicated that the twins have different biological fathers.

Although heteropaternal superfecundation is rarely observed among humans given its low frequency, in paternity disputes for dizygotic twins it is mandatory to demand the presence of the two twins in the testing to avoid wrong conclusions.

## Resumen

La superfecundación heteropaternal es un fenómeno extremadamente raro que se produce cuando un segundo óvulo, liberado durante el mismo ciclo menstrual, es fertilizado por un espermatozoide de un hombre diferente en relaciones sexuales separadas. En agosto de 2018, el Grupo de Genética de Poblaciones e Identificación de la Universidad Nacional de Colombia recibió una solicitud para establecer la paternidad mediante marcadores genéticos de un par de mellizos varones, en quienes se hizo el análisis del gen de amelogenina, el análisis de repeticiones cortas en tándem *(Short Tandem Repeats,* STR) autosómicas y del cromosoma Y (Y-STR) mediante kits comerciales de identificación humana y cálculos e interpretación del índice de paternidad y probabilidad de paternidad. Se obtuvo un índice de paternidad de 2,5134E+7 y una probabilidad de paternidad de 99,9999 % para el gemelo 2, en tanto que en el gemelo 1 se excluyeron 14 de los 17 marcadores del cromosoma Y y 14 de los 21 sistemas STR autosómicos evaluados. Los resultados indicaron que los gemelos tienen diferentes padres biológicos. A pesar de que la superfecundación heteropaternal rara vez se observa en humanos debido a su baja frecuencia, en las disputas de paternidad para los gemelos dicigóticos, es obligatorio exigir en la prueba la presencia de los dos gemelos para evitar conclusiones incorrectas.

Heteropaternal superfecundation is an extremely rare phenomenon that occurs when a second ova released during the same menstrual cycle is additionally fertilized by the sperm cells of a different man in separate sexual intercourse taking place within a short period of time from the first one ^1^^-^^4^. Wenk, *et al.,* found three cases in 39,000 records of a paternity-test database and showed a frequency of 2.4% heteropaternal superfecundation among dizygotic twins whose parents were involved in paternity disputes ^5^^,^^6^. Nevertheless, the frequency of these cases may vary depending on the population's coital rates and double ovulation rates ^3^.

This phenomenon was initially presented by Archer in 1810 ^7^; he showed the phenotypic differences between a white female twin and the other mulatto female twin and his study was later followed by Terasaki, *et al.,* in 1978 by typing histocompatibility antigens ^8^. Today, about 19 heteropaternal superfecundation cases worldwide have been reported ^2^^,^^4^^,^^7^^-^^23^. It has been suggested that this will cease to be a rare event, as cases are being and will be described more frequently thanks to the current availability of molecular methods and the popularity and increasing number of paternity tests ^2^^-^^4^^,^^14^^,^^22^.

In Colombia, Bravo-Aguilar presented a case of heteropaternal superfecundation as an example in the book "The genetic truth of the paternity" where two cases detected in the laboratory are mentioned, but no further information is given ^24^.

Here we present a case of dizygotic twins from different fathers detected in Colombia as evidenced by autosomal short tandem repeat (STR) DNA markers.

## Case presentation

In August, 2018, the *Grupo de Genética de Poblaciones e Identificación* at *Universidad Nacional de Colombia* received a request to establish the paternity of a pair of male twins with genetic markers from the alleged father, who suspected the children's kinship and requested paternity testing. Both twins were males, born after 35 weeks of gestation; the first male was 1,700 g and the second one, 2,380 g. The mother denied receiving blood transfusions during pregnancy, but she mentioned oral contraceptive intake before conception and antecedents of twins on her mother's side. The study was conducted with written consent from both the twins' mother and the alleged father. They also gave their consent for the publication of this work.

### Genetic profiles

Blood samples were collected from the twins, their mother, and the alleged father on FTA cards. We obtained two independent samples for each person taken at different times and each sample was investigated in duplicate.

DNA was extracted using two punches per sample and it was washed using FTA Reagent Purification buffer. To define the genetic profiles, the samples were typed for 15 autosomal STRs (D8S1179, D21S11, D7S820, CSF1PO, D3S1358, TH01, D13S317, D16S539, D2S1338, D19S433, vWA, TPOX, D18S51, D5S818, FGA) and amelogenin as the sex marker in the AmpFlSTR Identifiler Amplification Kit™ (Applied Biosystems).

To confirm and extend this analysis, we used 16 autosomal STRs (D10S1248, vWA, D16S539, D2S1338, D8S1179, D21S11, D18S51, D22S1045, D19S433, TH01, FGA, D2S441, D3S1358, D1S1656, D12S391, SE33) and amelogenin as the sex marker of the AmpFlSTR NGM SElect Kit™ (Applied Biosystems).

In another reaction, 17 Y-chromosome STR markers (DYS456, DYS389I, DYS390, DYS389II, DYS458, DYS19, DYS385a/b, DYS393, DYS391, DYS439, DYS635, DYS392, GATA H4, DYS437, DYS438, DYS448) were amplified simultaneously with the *AmpFlSTR Yfiler PCR Amplification Kit™* (Applied Biosystems).

These amplifications were done in an Applied Biosystems 2720 Thermal Cycler™. Capillary electrophoresis running and detection of amplified products were conducted with the ABI PRISM 310™ genetic analyzer. Samples were analyzed with GeneMapper ID™ software, version 3.2 (Applied Biosystem).

Alleged father - twin 1

Discrepancies in 14 autosomal STR markers and in 14 STR loci for Y chromosome (table 1) between the alleged father and twin 1 were observed.

Alleged father - twin 2

Results favored the paternity of the alleged father for only one of the twins whom we labeled as 'twin 2. All 21 autosomal and 17 Y-chromosomal markers were in concordance with paternity (table 1). The paternity index was 2.5134E+7.

**Table 1:** Genotypes of the mother, the alleged father, and the twins for 21 autosomal loci and genotypes of the alleged father and twins in 17 Y-chromosome loci

Autosomal STR					Y-STR			
System	Alleged father	Mother	Twin 1	Twin 2	Y-STR Loci	Alleged father	Twin 1	Twin 2
D8S1179	14 / 15	11 /13	11 / 12	13 / 15	DYS456	17	16	17
D21S11	32.2 / 32.2	30 / 30.2	27 / 30.2	30.2 / 32.2	DYS389I	13	12	13
D7S820	11 / 12	8 / 10	8/8	8/12	DYS390	24	21	24
CSF1PO	10	12	12 / 12	10 / 12	DYS389II	29	28	29
D3S1358	15 / 18	17	15 / 17	17 / 18	DYS458	16	15	16
TH01	6 / 7	6 / 9.3	7 / 9.3	7 / 9.3	DYS19	14	13	14
D13S317	12 / 13	10 / 11	10 / 11	10 / 13	DYS385a/b	11 / 14	13 / 16	11 / 14
D16S539	10 / 12	12	9 / 12	12	DYS393	13	14	13
D2S1338	17 / 23	17 / 22	22 / 22	22 / 23	DYS391	11	10	11
D19S433	13 / 14	13.2 / 13.2	12.2 / 13.2	13.2 / 14	DYS439	12	11	12
VWA	16 / 18	16 / 18	16 / 18	18	DYS635	23	22	23
TPOX	8	8 / 12	11 / 12	8 / 12	DYS392	13	15	13
D18S51	14 / 18	12 / 16	14 / 16	12 / 14	GATA H4	11	11	11
D5S818	11	11	11	11	DYS437	14	13	14
FGA	22 / 24	22	21 / 22	22 / 24	DYS438	12	12	12
D10S1248	15 / 16	14 / 15	13 / 15	14 / 16	DYS448	19	19	19
D22S1045	15 / 16	15 / 16	16	15 / 16				
D2S441	11 / 14	10 / 11	10 / 11	11				
D1S1656	11 / 12	17 / 17.3	17.3 / 17.3	12 / 17				
D12S391	16	17 / 26	17 / 18	16 / 17				
SE33	19 / 22.2	16 / 28.2	28.2 / 28.2	16 / 22.2				
Amelogenin	XY	XX	XY	XY				

As the genetic profile of the undoubted biological mother was available, the autosomal genotyping results indicated that the two twins had different fathers, which was confirmed with Y STR markers.

## Discussion

Few cases of heteropaternal superfecundation have been reported worldwide possibly because not all the cases have been involved in paternity disputes, which decreases the probability of their reporting ^3^^,^^5^^,^^22^. However, the frequency of cases will probably rise with time due to the availability of molecular techniques that serve as a tool for the correct resolution of such cases, as well as the recent increase in twin births ^2^.

In this case, the two brothers participated in the paternity study in response to a preview communication from the laboratory support service explaining that the test required the presence of both twins to study each of them separately, as well as the importance of including the mother. We observed 14 alleged father-twin 1 incompatibilities for Y-STRs loci (table 1) while no discrepancies between the alleged father and twin 2 were found allowing us to link twin 2 to the same paternal linage of the alleged father and to exclude twin 1 relationship to him. On the other hand, the analysis of the autosomal markers enabled us to establish the biological paternity of the alleged father in regard to twin 2 while excluding him as the father of twin 1. We also confirmed the biological maternity thus excluding any possibility of an interchange of the twins.

The difference between the birth weight of twin 1 and twin 2 was remarkable (680 g). This has also been mentioned by other authors ^4^^,^^10^^,^^12^^,^^22^ and is explained by the different gestational age of the children as a consequence of the time elapsed between one fertilization and the second one ^21^. Some authors assume that the time between the first and the second fertilization is about three or four days, although this time can be even longer (up to 14 days) ^1^^,^^5^^,^^6^.

Finally, this outcome allowed us to detect a case of dizygotic twins with different fathers, which is a rare and infrequent event reported in the literature. This case reiterates the need for the participation of both twins in paternity testing as these events happen although their frequency is not high.

## Conclusions

The results of the present study confirmed that these twins have different biological fathers.
